# Seismic Waveform Inversion Capability on Resource-Constrained Edge Devices

**DOI:** 10.3390/jimaging8120312

**Published:** 2022-11-22

**Authors:** Daniel Manu, Petro Mushidi Tshakwanda, Youzuo Lin, Weiwen Jiang, Lei Yang

**Affiliations:** 1 Department of Electrical and Computer Engineering, University of New Mexico, Albuquerque, NM 87106, USA; 2 Los Alamos National Laboratory, Bikini Atoll Road, SM-30, Los Alamos, NM 87545, USA; 3 Department of Electrical and Computer Engineering, George Mason University, Fairfax, VA 22030, USA; 4 Department of Information Sciences and Technology, George Mason University, Fairfax, VA 22030, USA

**Keywords:** full wave inversion, deep convolutional neural network, graphical user interface, data-driven method, structural similarity index metric, peak signal-to-noise ratio, Additive White Gaussian Noise

## Abstract

Seismic full wave inversion (FWI) is a widely used non-linear seismic imaging method used to reconstruct subsurface velocity images, however it is time consuming, has high computational cost and depend heavily on human interaction. Recently, deep learning has accelerated it’s use in several data-driven techniques, however most deep learning techniques suffer from overfitting and stability issues. In this work, we propose an edge computing-based data-driven inversion technique based on supervised deep convolutional neural network to accurately reconstruct the subsurface velocities. Deep learning based data-driven technique depends mostly on bulk data training. In this work, we train our deep convolutional neural network (DCN) (UNet and InversionNet) on the raw seismic data and their corresponding velocity models during the training phase to learn the non-linear mapping between the seismic data and velocity models. The trained network is then used to estimate the velocity models from new input seismic data during the prediction phase. The prediction phase is performed on a resource-constrained edge device such as Raspberry Pi. Raspberry Pi provides real-time and on-device computational power to execute the inference process. In addition, we demonstrate robustness of our models to perform inversion in the presence on noise by performing both noise-aware and no-noise training and feeding the resulting trained models with noise at different signal-to-noise (SNR) ratio values. We make great efforts to achieve very feasible inference times on the Raspberry Pi for both models. Specifically, the inference times per prediction for UNet and InversionNet models on Raspberry Pi were 22 and 4 s respectively whilst inference times for both models on the GPU were 2 and 18 s which are very comparable. Finally, we have designed a user-friendly interactive graphical user interface (GUI) to automate the model execution and inversion process on the Raspberry Pi.

## 1. Introduction

Seismic full-wave-inversion (FWI) has been widely used in subsurface geological exploration to describe rock quality, stratigraphic geology, energy exploration, etc. [1,2,3]. Specifically, FWI methods present information about subsurface stratas and rock geological properties where a 2D/3D velocity model is reconstructed from a given set of seismic observations. The mathematical implementation of FWI can be in the frequency domain or time domain [4,5,6,7]. In FWI, efficient seismic sources are used to produce seismic waves and the wave measurements are recorded via a seismometer. In order to solve the inversion problem, a forward model is used. The foward model characterizes how the seismic observations depend on the velocity model. The forward model can be either travel-time or FWI method. Travel-time inversion [8] is a simple technique which is based on linear estimation of the forward model, however they result in suboptimal inversion performance and resolution. FWI [9] techniques results in optimal inversion solutions but they are challenging due to non-linearity of the forward model, high computational cost and ill-posedness. The advantage of the FWI techniques is primarily their robustness to non-distributed data that result from the presence of noise and other factors. Several techniques have been proposed to solve the ill-posedness, which include: dynamic warping techniques [10], regularization-based methods [11,12,13], prior information-based approaches [14,15], multiscale inversion techniques [16] and precondition approaches [4]. High computational cost still remains a challenge when solving FWI problems. For instance, assuming there are *l* search steps required to find the best step at each iteration in a given 2D n×n velocity model, then the cost involved for the update will be $O(ln^{2})$.

With the recent achievements of deep neural networks (DNNs) [17,18] in image processing [19,20], data-driven seismic FWI techniques [21,22,23,24] have been developed which takes the seismic waveforms as input and then output the corresponding velocity models. Recently, Yang and Ma [25] proposed a supervised deep fully convolutional neural network (CNN) for velocity model reconstruction using raw seismic data. They fed their deep CNN model with multishot seismic data and their corresponding velocity models to learn the non-linear projection between them. During testing, they fed new input seismic data to the trained network to predict their velocity models. Their testing performance demonstrated that salt model prediction is much quicker and efficient using their technique compared to existing traditional and state-of-the-art techniques. Rojas-Gomez et al. [26] also developed a hybrid inversion technique that incorporated physics based models with data-driven methods. Their method consisted of an encoder-decoder network and an adaptive data augmentation technique. The data augmentation utilized the forward model to generate new training data that were more representative of the desired solutions. The authors demonstrated the performance by applying their inversion method to detect carbon sequestration leakage using synthetic datasets generated by subsurface model for CO${}_{2}$ storage site in Kimberlina, California. Their results yielded high accuracy and better generalization compared to the only data-driven and only physics driven techniques. Mao et al. [27] proposed a deep learning method for seismic exploration. They used CNN model which was fed with zero-offset multi-shot seismic data as input and the network outputs the velocity model. After training, the data assimulation process is driven to perform high precision inversion of the subsurface velocity. The authors tested their method on a designed salt body model and compared with traditional FWI techniques in high precision velocity inversion. Their results showed that the prior velocity model obtained by their method completes the velocity inversion under the high velocity body and successfully jumps out of the local minima caused by its inaccuracy.

Due to the extensive application of DNNs for data-driven seismic inversion, it becomes necessary to ensure the fast execution of DNNs on edge devices (e.g., Raspberry Pis, smartphones, Internet-of-Things (IoT) sensors) that are characterized by limited resources and real-time requirements for real-time velocity inversion. Moreover, edge computing techniques has been widely used for edge learning, which involves the deployment of DNN models (supervised, unsupervised, and reinforcement learning) at the network edge [28]. DNN computation on edge devices is challenging due to the limited resources however, these devices are excellent prospects for DNN execution due to their direct access to local raw data [29]. Due to computational requirements, DNNs are mostly executed on GPUs. However, modern edge devices are also equipped with high performance central processing units (CPU) or graphics processing units (GPU) which enable them to execute several small-scale to large-scale DNN models.

With the current state-of-the-art, inversion techniques are performed on very bulky high performance computing devices due to their high computational resources, however in this work, we have made great efforts and shown that inversion process can be successfully performed on portable and resource-limited devices such as Raspberry Pi within practical inference time without affecting the inversion performance.

## 2. Overview and Motivation

In this work, we present a seismic FWI approach based on the implementation of the shallow and deep DCN models on Raspberry Pi 4 to effectively reconstruct subsurface velocity models whilst achieving superior performance within an acceptable inference time. Currently, the seismic data collected by data operators are sometimes affected by interference such as noise and vibration effects which makes it very difficult to perform accurate inversion. Due to this, we are proposing an edge computing-based inversion technique. This is the first work to perform seismic inversion on a resource limited edge device. With the help of our system, it can provide data operators with real-time visualization and subsurface reconstruction capability for the data collected by the receivers on the field. With this, operators can be able to discard inferior data and collect only correct seismic data.

Our method includes two main phases: training and prediction phases. In the training phase, we feed the DCN models with seismic multishot gathers to learn the non-linear approximation between the seismic data and their equivalent velocity models. The training phase is completely performed offline on a GPU-based cluster due to limited resources on the Raspberry Pi device. In the prediction phase, the trained models are transferred to the Raspberry Pi to predict new velocity models from new unseen seismic data. Even though the training phase is computationally expensive which makes it difficult to execute on the Raspberry Pi, the cost involved during the prediction phase is very negligible on the Raspberry Pi. We demonstrate the robustness of our models to perform inversion even in the presence of noise by adding Additive White Gaussian Noise (AWGN) at different SNRs to our seismic data during training and testing. Here, we adopt two approaches: (1) Noise-aware model training and testing, where we train our DCN models on both clean and noisy data (0, 5, 10, 15, 20, 25 and 30 dB), and perform testing using both clean and noisy data, and (2) No-noise model training and Noise-aware testing where we train the models on only clean data, however we perform testing on both clean and noisy data.

In summary, the main contributions of our work include:

- We propose a novel edge computing-based inversion technique which is based on the implementation of DCN models on Raspberry Pi for inversion. Our DCN models are implemented based on modified versions of the UNet [30] and InversionNet [31] architectures. Both model architectures consist of convolution and deconvolution layers (encoder-decoder) which will be introduced in detail later in the paper.
- Our DCN models are implemented effectively on the Raspberry Pi to perform the inversion in real-time with superior performance.
- The inference times achieved for both models on the Raspberry Pi are very comparable to the inference times achieved on the GPU.
- We have designed a user-friendly and interactive GUI to automate and control the model execution and inversion process on the Raspberry Pi.

## 3. Background

In this section, we will first introduce a brief overview of the governing physics equation (acoustic-waveform inversion), physics-driven FWI and data-driven FWI methods.

### 3.1. Physics-Driven Full-Waveform Inversion

The physics-driven techniques estimate the subsurface velocity model using the governing physics and equations. Mathematically, the forward model can be expressed in terms of acoustic-wave equation using:    

(1) $$\begin{array}{c} m\;=\;\left[\frac{1}{K(r)}\frac{\partial^{2}}{\partial t^{2}}-\nabla \cdot \left(\frac{1}{\rho (r)}\nabla \right)\right]p\left(r,t\right)=s\left(r,t\right) \end{array}$$

where K(r) denotes the bulk modulus, ρ(r) refers to the density at spatial location *r*, p(r,t) is the pressure wavefield, *t* represents the time and · is the divergence operator. The above forward model in Equation (1) can be expressed as:

(2) $$\begin{array}{c} P\;=\;f(m) \end{array}$$

where *P* is the pressure wavefield in acoustic scenario or displacement wavefield in elastic scenario, *f* is the acoustic or elastic forward modeling operator and *m* refers to the velocity model parameter vector which includes the density and compressional, and shear-wave velocities. Given the forward model in Equation (2), the regularized seismic FWI can be expressed as:

(3) $$\begin{array}{c} m=\underset{M}{\mathrm{argmin}}\left\{{∥d-f\left(m\right)∥}_{2}^{2}+\lambda R\left(m\right)\right\} \end{array}$$

where *d* represents the recorded/field waveform dataset, f(m) is the equivalent forward modeling result, ${∥\cdot ∥}_{2}$ denotes the $L_{2}$-norm, ${∥d-f\left(m\right)∥}_{2}^{2}$ refers to the data misfit, λ is the regularization parameter, R(m) is the regularization term and argmin denotes the minimization term for the function. The commonly used regularization techniques are Tikhonov regularization and total-variation regularization. The Tikhonov regularization can be stated as:

(4) $$\begin{array}{c} E\left(y\right)=\min_{y}{∥x-f\left(y\right)∥}_{2}^{2}+\lambda ∥H_{y}{∥}_{2}^{2} \end{array}$$

where *H* denotes a high-pass filter or an identity matrix. Tikhonov regularization is mostly suitable for smooth models, hence waveform inversion with Tikhonov regularization results in blurred interfaces for piecewise-wise constant velocity models. The total-variation regularization was introduced in FWI to retain the sharp interfaces in the subsurface models. The current state-of-the-art physics-driven computation approaches used to reconstruct velocity models from their corresponding seismic data are based on gradient optimization techniques which is very computationally expensive and usually results in inferior resolution in detecting little structures.

### 3.2. Data-Driven Full-Waveform Inversion

The data-driven techniques converts the minimization problem in physics-driven methods to a mapping problem. Hence, the parameters of the velocity model (v) are learned using:

(5) $$\begin{array}{c} v\;=\;H\left(u\right)=f^{-1}\left(u\right) \end{array}$$

where $H=f^{-1}\left(\cdot \right)$ denotes the inverse operator of f(·), *v* is the subsurface velocity model and *u* represents the seismic data. The loss function can be defined as the minimization of the objective function below:

(6) $$\begin{array}{c} H=\underset{H}{\mathrm{argmin}}\sum_{i=1}^{N}{∥v_{i}-H\left(u_{i}\right)∥}_{2}^{2} \end{array}$$

where $\left(u_{i},v_{i}\right)$ represent the pairs of measured seismic observations and their equivalent velocity maps, and ${∥\cdot ∥}_{2}$ denotes the $L_{2}$-norm. Our data-driven approach directly obtains an estimation of $f^{-1}$ using a DCN which maps *u* to *v*. The DCN structure consists of an encoder-decoder architecture since our objective involves transforming data from one domain to another. The encoder extracts high-level features from the input data, thus reducing the dimension of the data, whilst the decoder transforms the extracted features into another domain based on our requirements.

## 4. Data and Model Description

This section presents the data preparation process which involves the design of the model (output) and data (input) for both the training and testing datasets. We use two datasets in this work namely SEG Salt and Kimberlina CO${}_{2}$ leakage datasets.

### 4.1. Salt Velocity Model Design

**Figure 1:**
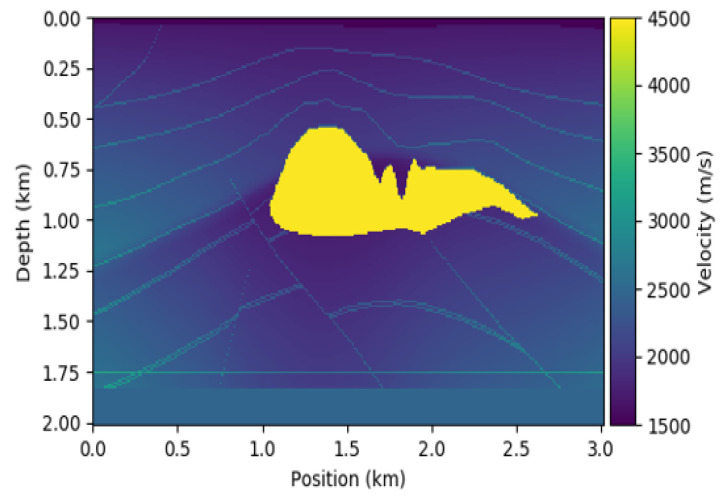
Sample velocity model of the 2D SEG Salt data.

In this work, we use the 2D SEG Salt velocity models. Each velocity model is assumed to have 5–12 layers as the base velocity with the velocity values from 2000 to 4000 m/s. Each velocity model has a salt body with random shape and position, and the salt body has a constant velocity of 4500 m/s. The shape of each velocity model is x×z=201×301 grid points with a spatial distance of Δx=Δz=10 m. We used 130 seismic data and their corresponding velocity models for the Salt SEG dataset (120 for the training set and 10 for the test set). Within the 120 training velocity models, 75 of them have the salt structure whilst the remaining 45 have no salt. Figure 1 shows a sample velocity model of the 2D SEG Salt data.

### 4.2. Salt Data Design

**Figure 2:**
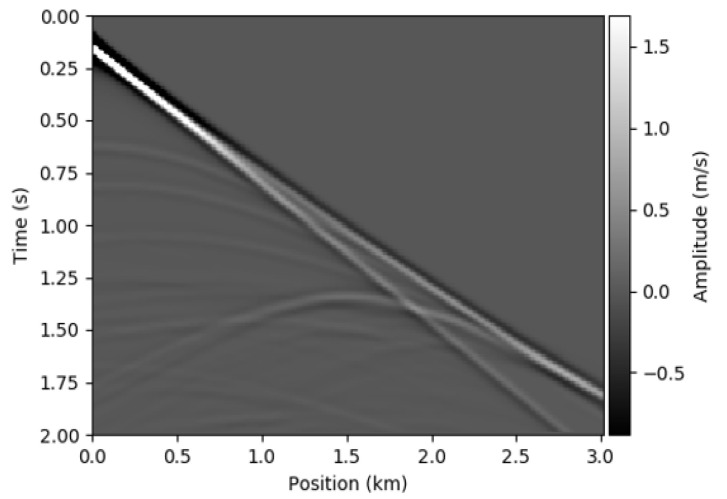
Sample seismic data of the 2D SEG Salt data.

Here, we used 29 sources evenly placed to generate the seismic shot gathers and then 301 receivers evenly placed at regular intervals are used to record the seismic measurements. During the training phase, we used a batch size of 3 random samples of velocity models from the training set and for each batch of training data, we downsampled one-shot gather data to 200×301 using a downsampling ratio of 1:1. Figure 2 shows a sample seismic data of the 2D SEG Salt data.

### 4.3. Kimberlina Data and Velocity Model Design

**Figure 3:**
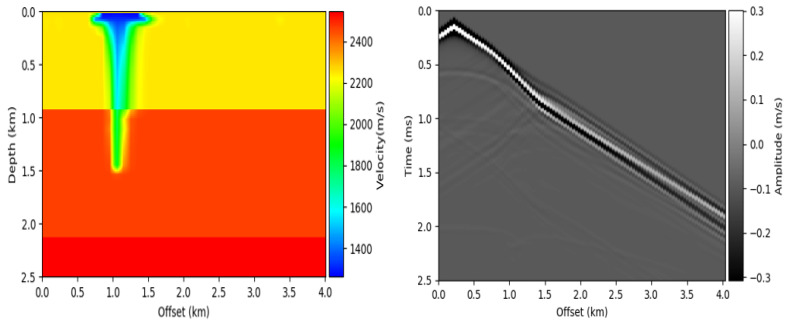
Sample velocity model (**left**) and seismic data (**right**) of the Kimberlina data.

We used the simulated Kimberlina dataset from Lawrence Livermore National Laboratory. The kimberlina dataset helps to understand and evaluate the performance of different geophysical monitoring methods in detecting CO${}_{2}$ shallow leakage in a wellbore [32]. This helps to obtain major reductions in atmostpheric CO${}_{2}$. The Kimberlina CO${}_{2}$ characterizes the spatial and temporal movement of a critical CO${}_{2}$ plume within a reservoir, containing 991 CO${}_{2}$ leakage cases that are simulated over a period of 200 years with 10 leakage maps given for each case. The Kimberlina dataset is created through a commercial-scale geological carbon sequestration (GCS) reservoir at the Kimberlina site in the southern San Joaquin Basin, 30 km northwest of Bakersfield, CA, USA. The synthetic seismic data is obtained from the CO${}_{2}$ leakage velocity models via forward modeling. Each velocity model has a randomly shaped plume of a constant velocity. The shape of the velocity model is x×z=141×401 grid points with spatial distance of Δx,Δz=0.0177 m, 10 m. The seismic data is generated using 9 sources and 101 receivers evenly distributed at regular intervals. The shape of the seismic data is x×y×z=9×1251×101 with spatial distance of Δy,Δz=0.002 s, 40 m. We used 130 seismic data and their corresponding velocity models for the Kimberlina dataset (120 for the training set and 10 for the test set). Figure 3 shows a sample velocity model (left) and seismic data (right) of the Kimberlina data.

### 4.4. DCN Architecture

In this work, our DCN architecture is implemented based on deep convolutional layers. Specifically, we modified the UNet and InversionNet architectures to be compatible with the input seismic data dimensions. Both architectures consist of the encoder and decoder. The encoder comprises of a set of convolution blocks which contains convolution layers, batch normalization [33] and ReLU [34]. The convolution layers convolve the input seismic data by using filters to extract relevant features. Batch normalization is used to normalize the inputs (zero-means, unit variance and decorrelated) to a layer for every mini-batch. This stabilizes the training process and makes the network converge much faster. The ReLU activation function accounts for non-linearities by assigning zero to negative input values. The decoder consists of a mixture of convolution and deconvolution blocks. The deconvolution block, also known as the transposed convolution expands the size of its input by padding zeros on the input feature maps.

**Figure 4:**
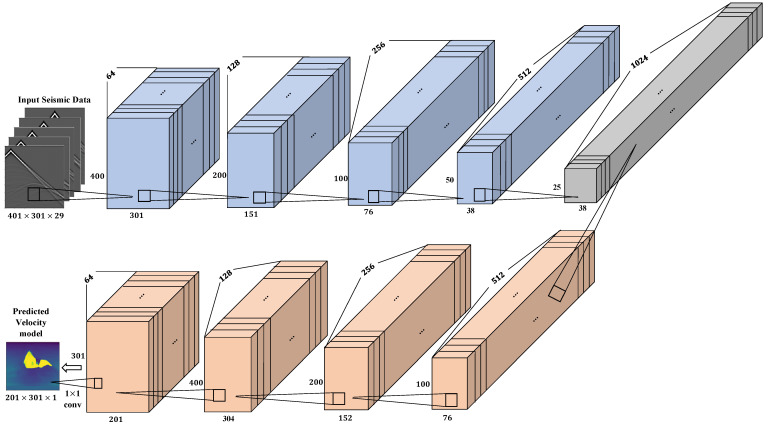
Modified UNet architecture used for the seismic inversion.

Modified UNet: Each convolutional layer in the UNet uses a fixed kernel size of 3×3. The channel dimensions used in the convolution layers are 64,128,256,512 and 1024, as the network depth increases. After the convolutions, the max pooling layer of kernel size 2×2 with stride 2 is applied to reduce the shapes of the feature maps to half of their previous shape. Deconvolution layers with the same channel dimensions as the convolution layers are applied to the feature maps to expand the shape of the output feature maps to be the same as the input. Specifically, we used a fixed kernel size of 5×5 with stride 2 in the deconvolution layers. Finally, the soft-max function is used to obtain the predicted label. The predicted label shows the pixels that belong to the salt body within the seismic data. We used the mean squared error (squared $L_{2}$ norm) to compute the loss between the predicted label and ground truth label which is defined by:    

(7) $$\begin{array}{c} L_{2}\left(y_{g},y_{p}\right)\;=\;\sum_{i=1}^{n}{\left|y_{pi}-y_{gi}\right|}^{2} \end{array}$$

where $y_{g}=\left\{y_{g1},\ldots ,y_{gn}\right\}$ is the ground truth, $y_{p}=\left\{y_{p1},\ldots ,y_{pn}\right\}$ is the predicted velocity model and *n* is the number of spatial locations in the velocity model. Figure 4 shows the architecture of the UNet used for the seismic inversion. Mathematically, the operations in the UNet can be represented by the expression below:

(8) $$\begin{array}{c} y\;=\;\mathrm{UNet}\left(x;\Theta \right)=S(K_{2}*\left(P\left(\alpha \left(K_{1}*x+b_{1}\right)\right)\right)+b_{2}) \end{array}$$

where UNet() represents non-linear mapping of the network, *x* denotes the input, *y* is the output, $K_{1},K_{2}$ are the convolutional kernel dimensions, $b_{1},b_{2}$ are the biases, $\Theta =\{K_{1},K_{2},b_{1},b_{2}\}$ is the set of parameters to be learned, α denotes the activation function such as the Rectified Linear Unit (ReLU), sigmoid, etc., *P* denotes the pooling function (e.g., max-pooling), “∗” represents the convolution operation, S() denotes the soft-max function. Since our approach is based on supervised learning, the network has to be fed with input-output pairs (seismic data and their corresponding velocity models). Given that our aim is to predict the velocity models using the seismic data, the UNet model learns a non-linear mapping between the seismic data (input) and their corresponding velocity model (output). Hence, the model projects the seismic data from the data distribution to model distribution. The network learns by solving the objective function below:

(9) $$\begin{array}{c} \hat{\Theta }=\underset{\Theta }{\mathrm{argmin}}\frac{1}{pN}\sum_{n=1}^{N}L_{2}\left(v_{n},\mathrm{DCN}\left(d_{n};\Theta \right)\right)\\ \mathrm{for}\;{\tilde{v}}_{n}\;=\;\mathrm{DCN}\left(d_{n};\Theta \right) \end{array}$$

where *p* denotes the total number of pixels in a velocity model, $L_{2}\left(\cdot \right)$ is the error value between the ground-truth values $v_{n}$ and predicted values ${\tilde{v}}_{n}$. In order to update the learned parameters, Adam and back-propagation algorithms are used. The Adam optimizer [35] updates the parameters iteratively using:

(10) $$\begin{array}{c} \Theta_{t}=\Theta_{t-1}-\alpha \cdot {\hat{m}}_{t}/\left(\sqrt{{\hat{v}}_{t}}+\epsilon \right) \end{array}$$

where ${\hat{m}}_{t}\leftarrow m_{t}/\left(1-\beta_{1}^{t}\right)$, ${\hat{v}}_{t}\leftarrow v_{t}/\left(1-\beta_{2}^{t}\right)$, α is the positive step size, $\epsilon \;=\;10^{-8}$, $\beta_{1}$ and $\beta_{2}$ have their default values of 0.9 and 0.999 respectively as used in the Adam paper.

Modified InversionNet: In this architecture, the encoder is implemented with a stack of 14 convolution layers with the first layer having a kernel size of 7×1 and the next six layers having a kernel size of 3×3. We used a stride of 2×1 in the first convolution layer to reduce the data dimension to the velocity model dimension. The six convolution layers with kernel size 3×3 are used to extract spatial-temporal features in the data where a stride of 2 is used to downsample the data in each layer. Next, a convolution layer with kernel size 10×2 is used to flatten the feature maps to an output latent vector dimension (512 in this case). The decoder comprises of first deconvolution layer with kernel size 5×13 which is applied on the latent vector to produce a 5×13×512 tensor followed by a convolution layer with same input and output channel dimensions. After the first deconvolution layer, series of deconvolution-convolution operations are performed with kernel sizes of 4×4 and 3×3 in the deconvolution and convolution layers respectively. Finally, we use the negative padding technique with pad dimensions [−7,−8,−9,−10] to crop the feature maps and apply a 3×3 convolution layer to get an output of a single velocity map of shape (141×401). Both the convolution and deconvolution layers are followed with batch normalization and LeakyReLU activation function. The $L_{1}$ loss function is used to compute the reconstruction error, which is given below:

(11) $$\begin{array}{c} L_{1}\left(x,y\right)\;=\;\frac{1}{n}\sum_{i=1}^{n}\left|x_{i}-y_{i}\right| \end{array}$$

where $y\;=\;\{y_{1},\ldots ,y_{n}\}$ is the ground truth, $x\;=\;\{x_{1},\ldots ,x_{n}\}$ is the predicted velocity model and *n* is the number of spatial locations in the velocity model.

**Figure 5:**
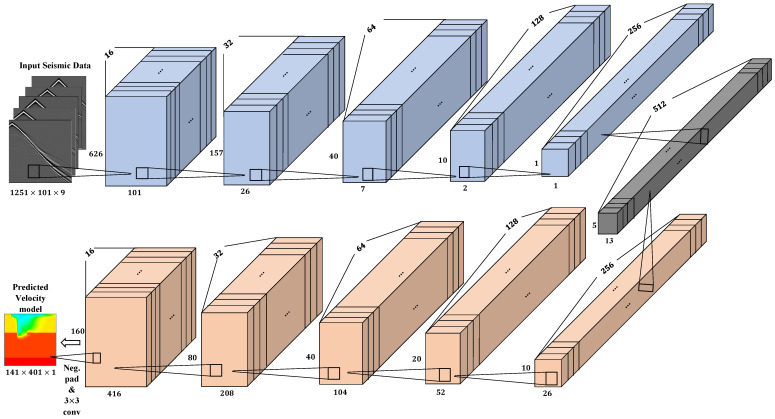
Modified InversionNet architecture used for the seismic inversion.

Our modified InversionNet architecture consists of 14 CNN layers in the encoder and 13 layers in the decoder. Figure 5 shows the architecture of the InversionNet model.

## 5. Noise Addition

As stated earlier, we employed two techniques for the noise addition: Noise-aware training and testing, and No-noise training and noise-aware testing. We used the SNR method to generate the noise which is given by Equation (12). SNR refers to the measure of the power of the desired signal relative to the background noise. Higher values of SNR results in a better image output. We used the SNR approach to precisely evaluate our noisy seismic image quality, because high SNR is always required in modern vision applications which involve edge-based processing where ML models are used to study the processed images.

(12) $$\begin{array}{c} \mathrm{SNR}\left(\mathrm{dB}\right)\;=\;10\log_{10}\frac{P_{\mathrm{image}}}{P_{\mathrm{noise}}} \end{array}$$

where $P_{\mathrm{image}}$ is the image power in watts and $P_{\mathrm{noise}}$ is the noise power. We generated noise at 7 different levels (i.e., 5, 10, 15, 20, 25 and 30 dB). For noise-aware training, we generated 120 seismic data at each of these noise levels in addition to the clean data and fed all to the DCN model. During testing, we fed a noisy data at any of these noise levels to the trained models to predict the velocity model. The noise addition process as depicted in Algorithm 1 starts by first flattening the seismic image data. The image power (watts) is then computed by taking the square of all the image pixel values. The average image power (watts) is computed by taking the mean of all squared pixel values. The average image power (watts) is converted to its dB equivalent using Equation (13).

(13) $$\begin{array}{c} \mathrm{Avg}.P_{\mathrm{image}}\left(\mathrm{dB}\right)\;=\;10\log_{10}\frac{\mathrm{Avg}.P_{\mathrm{image}}\left(\mathrm{watt}\right)}{1\mathrm{watt}} \end{array}$$

The average noise power in dB is computed by subtracting the set target SNR value (dB) from the average image power in dB as shown in Equation (14). The average noise power (dB) is then converted to its watt equivalent using Equation (15).

(14) $$\begin{array}{c} \mathrm{Avg}.P_{\mathrm{noise}}\left(\mathrm{dB}\right)\;=\;\mathrm{Avg}.P_{\mathrm{image}}-\mathrm{Target}\;\mathrm{SNR} \end{array}$$

(15) $$\begin{array}{c} \mathrm{Avg}.P_{\mathrm{noise}}\left(\mathrm{watt}\right)\;=\;10^{\frac{\mathrm{Avg}.P_{\mathrm{noise}}\left(\mathrm{dB}\right)}{10}} \end{array}$$

**Figure 6:**
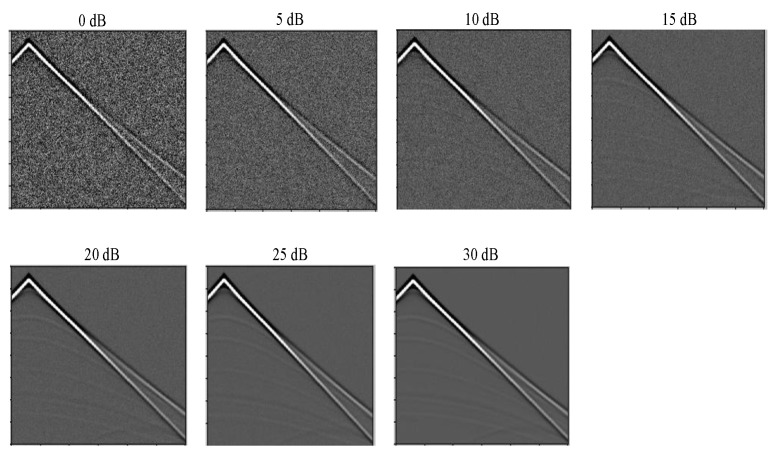
Seismic image representation at each noise level.

**Figure 7:**
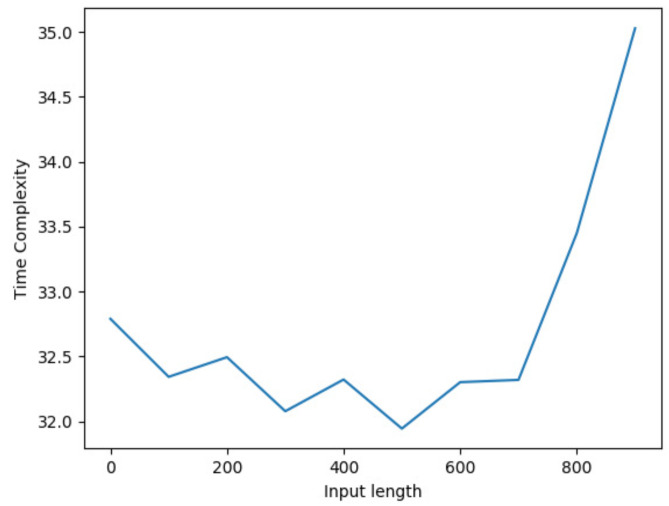
Illustrative time complexity plot for the noise algorithm.

Additive White Gaussian Noise (AWGN) of length equal to the flattened image is generated. In order to model the AWGN, a zero-mean Gaussian random variable was added to the seismic image. Hence, it should be noted that the mean (μ) of the generated AWGN should be equal to zero and the standard deviation (σ) should be equal to the square root of the average noise power. The variance of the random variable will then affect the average noise power. For instance, given a Gaussian random variable *X*, the average power $E[X^{2}]$, which is also known as the second moment is given by $E\left[X^{2}\right]=\mu^{2}+\sigma^{2}$, where $\sigma^{2}$ is the variance. The generated noise is then added to the original flattened image and finally reshaped back to the original image shape. Figure 6 shows the image of seismic data at each noise level. The performance of the noise algorithm was analyzed in terms of time complexity. Specifically, O(n) time is involved during the flattening the seismic image, O(n) time for computing image power (watts), O(n) time for computing average image power (watts), O(1) time for computing average image power (dB), O(1) time for computing average noise power (dB), O(1) time for converting average noise power from dB to watts, O(n) time for generating AWGN, O(n) time for adding generated noise to image and finally O(n) time for reshaping noisy image to original image shape. Hence, the resultant time complexity for the noise addition algorithm is linear time O(n) which is greatly acceptable. Figure 7 shows an illustrative time complexity plot for the noise algorithm for input length up to 1000. It can be seen from the figure that the time complexity reduces for input length 0 up to input length 700 and starts increasing linearly after input length 700 which demonstrates the linear time complexity of the noise algorithm (see Algorithm 1) for increasing input length.
**Algorithm 1** Algorithm to add noise to the seismic image.**Require:** Inputseismicimage**Ensure:** Flattenseismicimageto1−D**Ensure:** SettargetSNRvalue.e.g.,SNR=10dB  ☞**Step 1** Compute image power in watts (square all image pixels)  ☞**Step 2** Compute average image power in watts (mean of all squared image pixels)  ☞**Step 3** Compute average image power in dB (use Equation (13))  ☞**Step 4** Compute average noise power (dB) (use Equation (14))  ☞**Step 5** Convert average noise power from dB to watts (use Equation (15))  ☞**Step 6** Generate AWGN (μ=0 and $\sigma \;=\;\sqrt{\mathrm{Avg}.P_{\mathrm{noise}}}$) with length equal to flattened image  ☞**Step 7** Add generated noise to original flattened image  ☞**Step 8** Reshape resulting image back to original shape

## 6. User Interactive GUI Design

**Figure 8:**
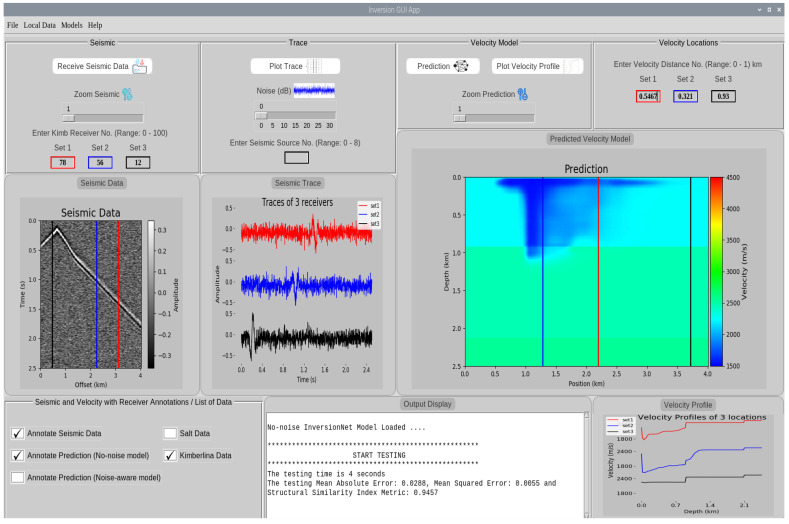
Interface of the designed graphical user interface.

In order to automate and control the inversion process on the Raspberry Pi, we designed an interactive and user-friendly GUI application where the user can interact to perform the inversion on the Raspberry Pi. Figure 8 shows the interface of the designed GUI. The GUI incorporates different functionalities such as receiving the seismic data, showing the predicted velocity model, plot seismic traces of receivers and velocity profiles at specific locations, noise addition as well as zooming in and out options for the received seismic data and the predicted velocity models. The various parts of the GUI application are described below:

**Seismic Data:** Here, the user can receive a randomly sampled test data from the locally stored data. We also provide an option for the user to select and receive a specific test data of interest to perform inversion on by selecting **Local Data** on the menu bar. By default, all the received seismic data are from the second channel (source), however we have provided an alternative for users to receive data from a specific source using the **Enter Seismic Source No.** input. The receive seismic data button displays the randomly sampled or user selected data.

**Trace:** The trace provides information about the signals received by the receivers. By default, we plot the trace of receivers 50, 150 and 250 for the Salt data and 30, 60 and 90 for the Kimberlina data. The user can also give different receiver numbers using the **Set 1–3** input to plot the user-defined traces.

**Velocity Model:** The velocity model section comprises of the prediction and velocity profile. The user can perform prediction on the received test data using a specific DCN model and plot the velocity profiles at different locations or distances. The velocity profile gives information about the signals occurring at a specific location within the predicted velocity model. The location ranges for Salt and Kimberlina data are **0–3** km and **0–1** km respectively.

**Model Selection:** The various models used in this work are located under **Models** in the menu bar. Specifically, we used four models namely: No-noise UNet, Noise-aware UNet, No-noise InversionNet and Noise-aware InversionNet models.

**Output Display:** The output display shows the inference performance, time spent for prediction, data and model that have been loaded by the user. In this work, we measure the inference performance for Salt data in terms of structural similarity index measure (SSIM) and peak signal-to-noise ratio (PSNR) whereas we measure mean absolute error (MAE), mean squared error (MSE) and SSIM for Kimberlina data.

For instance, from Figure 8, we can observe that the received seismic data is from the second channel with noise 0 dB. Annotations for receivers 78, 56 and 12 are shown on the seismic data which corresponds to offset values of 3.12 km (red), 2.24 km (blue) and 0.48 km (black) since 1 pixel on the offset corresponds to 40m. The traces for receivers 78, 56 and 12 are shown in the trace section. The prediction for the received data is shown in the velocity model section. It can be observed that the model made a good prediction even though the seismic data is very noisy. Annotations for velocity locations 0.5467, 0.321 and 0.93 are shown on the velocity model which corresponds position values of 2.1868 km (red), 1.284 km (blue) and 3.720 km (black). The velocity profiles corresponding to these locations are also shown in the velocity profile section. On the output display, it can be seen that the prediction was made using the no-noise InversionNet model. The prediction time involved is recorded as 4 s whilst the testing MAE, MSE and SSIM are recorded as 0.0270, 0.0051 and 0.9476 respectively.

## 7. Experimental Setup and Results

### 7.1. Training Settings

As mentioned earlier, we conducted the DCN training on a Tesla K40m GPU with 12 GB memory. During training of the UNet, we fed 120 and 960 Salt data and their corresponding velocity models to the network for no-noise and noise-aware training respectively. A batch size of 3 is used during training and network is trained for 500 epochs. We used a learning rate of 0.001 during training and models are saved at each 20 epochs. The training times involved were approximately 1 h 30 min and 8 h for no-noise and noise-aware training respectively. For InversionNet, we fed 120 and 960 Kimberlina data and their velocity models for no-noise and noise-aware training respectively. A batch size of 10 is used and network is trained for 500 epochs. A learning rate of 0.0001 is used and models are saved at each 50 and 100 epochs for no-noise and noise-aware training respectively. The training times involved for no-noise and noise-aware training were 1 h 30 min and 9 h respectively.

### 7.2. Inference Performance

**Figure 9:**
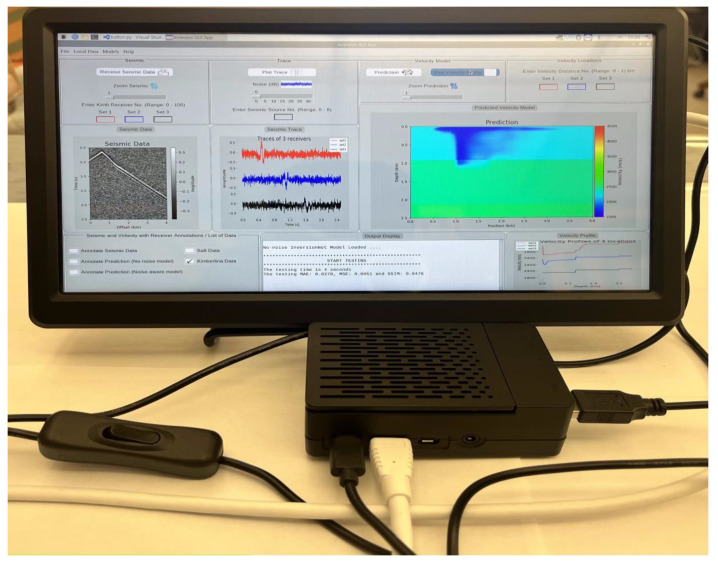
Raspberry Pi setup for model execution and inversion.

After training, the saved models are transferred to the Raspberry Pi for inference. Figure 9 shows our Raspberry Pi setup used for the inversion process. The Raspberry Pi used in our work has the following specifications: Quad core Cortex-A72 @ 1.5 GHz and 8 GB RAM. We used 10 Salt and Kimberlina data for the inference. During the inference, we used a batch size of 1 where one random data is selected or user selects a specific data from the test dataset and then the velocity model for that data is predicted by the model. We recorded the inference performance in terms of SSIM and PSNR for Salt data and measure MAE, MSE and SSIM for Kimberlina data. PSNR measures the quality between ground truth and predicted velocity images. The higher the PSNR value, the better the quality of the predicted velocity image. SSIM measures the similarity between ground truth and predicted velocity images. The SSIM ranges from 0 to 1, and higher values are better. MSE measures the average squared difference between the predicted and ground truth. MAE measures the average absolute error between the ground truth and predicted values. The lower the MSE and MAE values, the more accurate the model is. Table 1 shows the comparison of inference performances (PSNR and SSIM) for all the 10 clean Salt test data for no-noise and noise-aware UNet models respectively. From the table, we can see that the no-noise models outperformed the noise-aware models in most of the test data namely: sample 2 (PSNR, SSIM = 17.9765,0.5340 vs. 17.8331,0.5338), sample 3 (PSNR, SSIM = 12.9452,0.5110 vs. 14.4647,0.4907), sample 5 (PSNR, SSIM = 26.2139,0.5325 vs. 24.2624,0.4877), sample 6 (PSNR, SSIM = 22.5901,0.5032 vs. 16.1032,0.4251), sample 7 (PSNR, SSIM = 22.7110,0.4629 vs. 19.2977,0.4159) and sample 8 (PSNR, SSIM = 15.8107,0.5168 vs. 14.9872,0.4984) whilst noise-aware models outperformed the no-noise models in sample 1 (PSNR, SSIM = 16.0833,0.5169 vs. 16.3088,0.4873), sample 4 (PSNR, SSIM = 16.4363,0.2779 vs. 13.4861,0.2539) and sample 10 (PSNR, SSIM = 18.0979,0.5965 vs. 13.9382,0.5091). Table 2 shows the comparison of inference performances (SSIM) for all the 10 clean Kimberlina test data for no-noise and noise-aware InversionNet models respectively. From the table, the noise-aware models outperformed the no-noise models in all the test data except sample 4 (SSIM = 0.983 vs. 0.983), sample 6 (SSIM = 0.993 vs. 0.992) and sample 9 (SSIM = 0.999 vs. 0.969).

### 7.3. Inference Time

With the execution of the inference on the Raspberry Pi which has limited resources in terms of computational power and memory requirements, it is very necessary to ensure that the time taken for one prediction is within acceptable limits. For our experiments, we observed that the time taken to make one prediction on the Raspberry Pi for Salt and Kimberlina data were around 22 and 4 s respectively, which is very feasible and acceptable. We compared our results with the inferences times achieved by performing inference on GPU PC with specifications listed in Table 3. From the results in Table 3, running InversionNet on Raspberry Pi obtains a smaller inference time (4 s) compared to 18 s on the GPU PC. However, for UNet, the inference time on the GPU PC outperformed that of the Raspberry Pi. We made great efforts to optimize our models to perform inversion within feasible inference times on the Raspberry Pi. For instance, we achieved 4.5× lower inference time on the Raspberry Pi compared to the GPU for the deep InversionNet model.

### 7.4. Reconstruction Results

**Figure 10:**
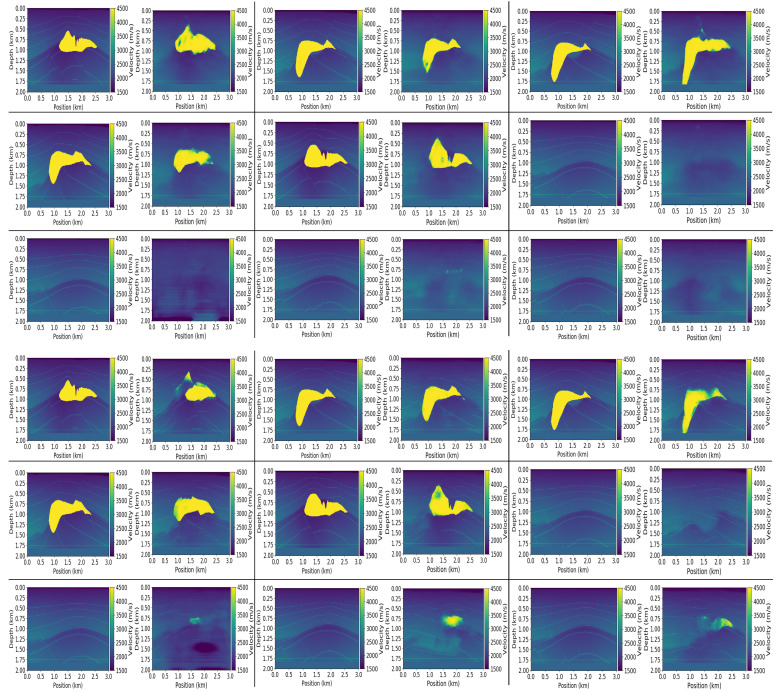
Reconstruction results for no-noise (**top**) and noise-aware (**bottom**) models on the Salt data. Ground truth (**left**) and Prediction (**right**).

**Figure 11:**
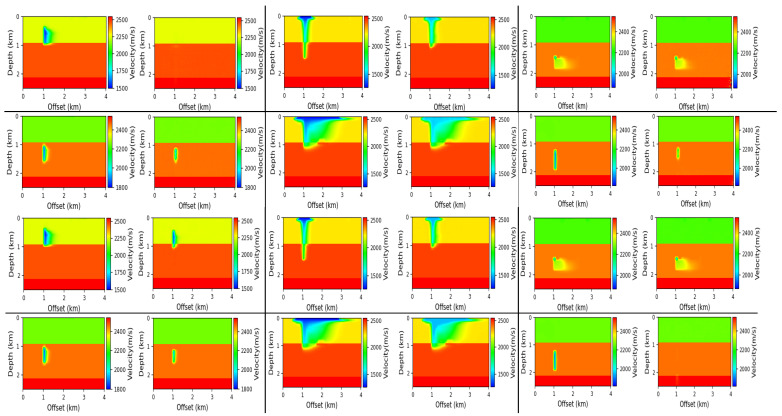
Reconstruction results for no-noise (**top**) and noise-aware (**bottom**) models on the Kimberlina data. Ground truth (**left**) and Prediction (**right**).

Figure 10 shows some reconstruction results for no-noise and noise-aware models on Salt data. We can observe from the results that both models are able to make very good predictions. Comparing the no-noise and noise-aware results by visual inspection, we can see that no-noise models made better predictions in the top left (SSIM = 0.5110 vs. 0.4907), top mid (SSIM = 0.5340 vs. 0.5338), mid left (SSIM = 0.5168 vs. 0.4984), mid right (SSIM = 0.5325 vs. 0.4877), bottom mid (0.5032 vs. 0.4251) and bottom right (0.4629 vs. 0.4159) reconstructions compared to the noise-aware models. These imperfect predictions by the noise-aware models resulted because of overfitting of the models to the training data since more data were used for training the noise-aware models. Because the UNet model is a shallow network with few layers (4 layers), the noise-aware models learn all the unnecessary details within the data which negatively affects the generalization performance on the test data. Notwithstanding that, the noise-aware models made better predictions in the top right (SSIM = 0.5965 vs. 0.5091), mid middle (SSIM = 0.5169 vs. 0.4873) and bottom left (0.2779 vs. 0.2539) reconstructions. Figure 11 shows some reconstruction results for the Kimberlina data. In the case of Kimberlina dataset, the reconstruction results for noise-aware models were quite better than the no-noise models. Specifically, by visual inspection, we can observe that the noise aware models made better prediction for top right (SSIM = 0.991 vs. 0.984), top mid (SSIM = 0.981 vs. 0.978), top right (SSIM = 0.999 vs. 0.995), bottom left (SSIM = 0.998 vs. 0.997) and bottom mid (SSIM = 0.961 vs. 0.942) reconstructions. The no-noise models made a better prediction than the noise-aware models in only the bottom-right (SSIM = 0.993 vs. 0.992) reconstruction. The noise-aware models outperformed the no-noise models for the Kimberlina data because the InversionNet model is a deep network with more layers (27 layers) and hence need more data to learn most of the feature representations. Since the noise-aware model is trained with more data (960 in this case) compared to only 120 for the no-noise model, the noise-aware models can learn more feature representations in the seismic data hence performs better than the no-noise models.

### 7.5. Effects of Noise at Different SNRs on Reconstructions

**Figure 12:**
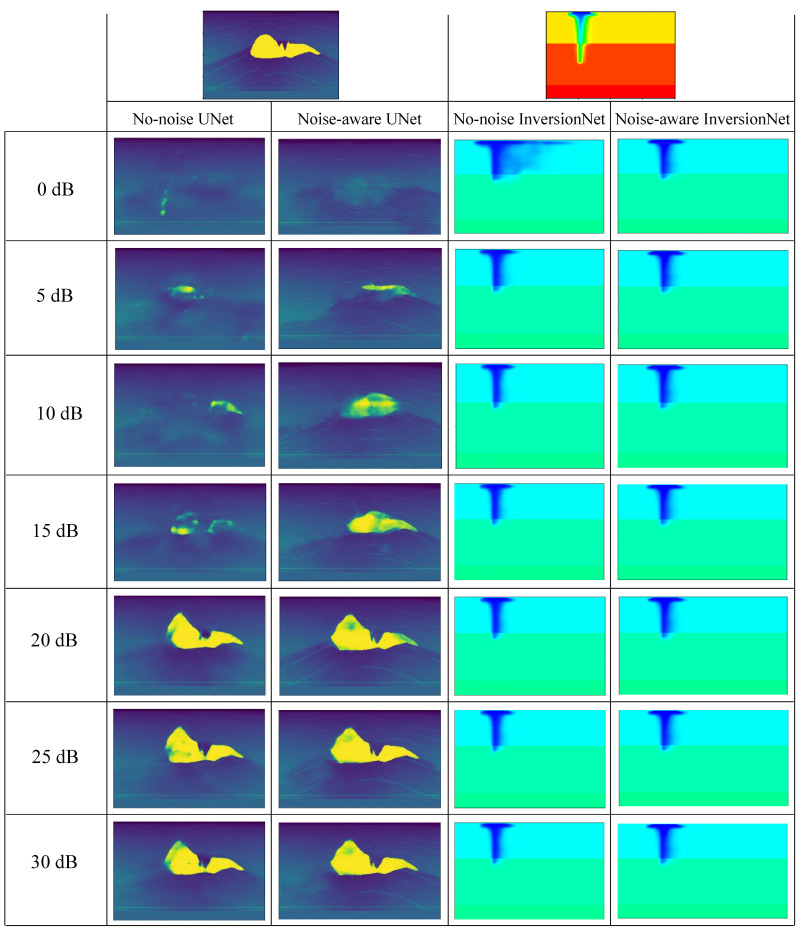
Effect of noise at different SNRs on reconstructions predicted by no-noise and noise-aware models.

In order to demonstrate the effects of different noise on reconstructions predicted by both no-noise and noise-aware models, we conducted extensive experiments with results displayed in Figure 12. Starting from the left with the reconstructions from the no-noise UNet model, we can observe that adding noise with low SNR (e.g., 0 dB) resulted in poor reconstructions (no salt detection). As the SNR value was gradually increased, the quality of the reconstructed image started improving and detecting the salt structure. At 30 dB, we can say that the noise component in the seismic data is very minimal, hence the reconstructed image was good compared to the ground truth. Comparing reconstructions from the no-noise and noise-aware UNet models, we can observe from the results that the noise-aware reconstructions were better than the no-noise, which is very accurate. This is due to the fact that the noise-aware models were trained on data from each of these noise levels hence the model can produce a good reconstruction on any data affected with any of these noise levels. For inversionNet, the reconstruction performance for both no-noise and noise-aware models were very comparable to the ground truth except only at 0 dB where the reconstruction of the no-noise model was slightly different from the ground truth. As stated earlier, the inversionNet has more layers hence the no-noise model can even learn significant features from the clean data and translate it to noisy data to produce good reconstructions. For noise-aware InversionNet, we can see from the results that all the reconstructions were very similar to the ground truth at each noise level. Therefore from the results, we can conclude that the noise-aware models were more robust to the noisy data at different SNRs compared to the no-noise models, which is expected.

### 7.6. Scalability of Datasets and Proposed Method to Real-World/Field Applications

Our proposed technique can be applied in a wide range of applications depending on the input seismic data. Some of the applications include subsurface characterization to determine rock quality and geological nature of a specific site, and detect ground water contamination, etc. For instance, in subsurface characterization, data operators collecting data on the field can connect our designed system (edge device—Raspberry Pi, tablet, cell phone, etc.) to the field data receivers via bluetooth, wireless or network cable to visualize the real-time subsurface inversion reconstructions of the collected data. This can enable these data operators to discard contaminated and inferior data in real-time based on the subsurface inversion output.

Our synthetic dataset is affected by the parameters (e.g., model grid spacing, number of sources and receivers used, source and receiver interval distance, etc.) used to generate them. In this work, we generated the synthetic salt data with the parameters stated under Section 4.1 and Section 4.2 by applying forward modeling on the velocity models based on the forward-propagated source wave equation. For Kimberlina dataset, we used the simulated data generated based on the hypothetical numerical model built on the geological structure of the GCS reservoir at the Kimberlina site. The P-wave and S-wave velocity maps used in this work originated from the geophysical model built on the realistic geological-layer characteristics from the GCS site. Since our models are trained on datasets with these specifications, to scale our datasets and proposed techniques accurately to the real-time field data, the field data should be collected using similar parameters used in this work for our models to effectively generate optimal subsurface reconstructions. However, if the field setup used for the data collection has different configuration compared to our assumed synthetic data generation parameters, samples of the field data can be collected and trained on our models and then the trained models can be applied on the field data to obtain the subsurface reconstructions in real-time. Moreover, since our training velocity models are diverse (i.e., with salt and without salt structures), our models are able to learn the mapping of the seismic data to their corresponding velocity models with or without salt structures, hence our trained can produce good reconstructions on real-time field data whose velocity models have salt or no salt.

### 7.7. Comparison of Our Results with Existing Works

**Figure 13:**
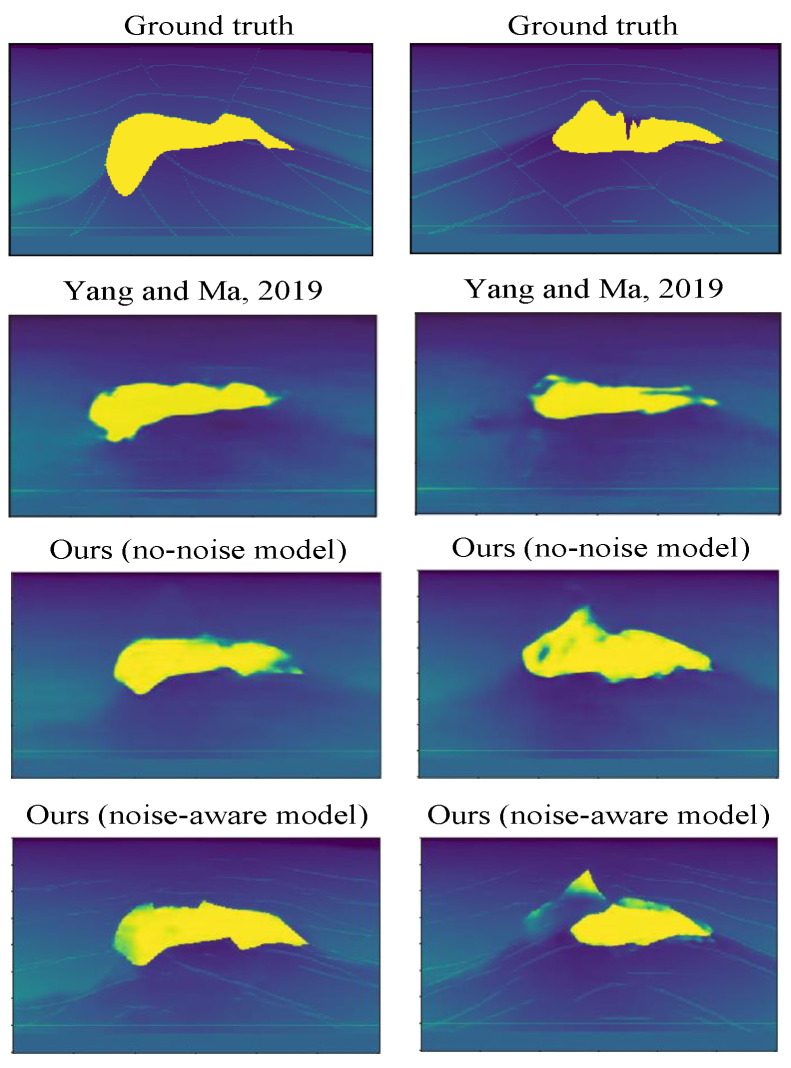
Comparison of reconstructions between our work and Yang and Ma [25].

In this section, we compare our proposed methods to the existing works such as Yang and Ma [25]. In their work, the authors performed both training and testing on an HP Z840 workstation with a Tesla K40 GPU, 32 Core Xeon CPU, 128 GB RAM. In order to demonstrate the robustness and stability of their method, they added a fixed Gaussian noise with zero mean and standard deviation of 5% to each of the testing data. In their work, the total time involved for training 130 SEG salt data was 43 min. The inference time for each seismic data was 2 s. However in our work, we performed training on Tesla K40m GPU with 12 GB RAM and the trained models were used to perform testing on the Raspberry Pi with these specifications: Quad core Cortex-A72 @ 1.5 GHz and 8 GB RAM. In our work, we performed both no-noise training (similar to theirs) and noise-aware training. The time involved for training our 120 seismic data without noise was 1 h and 30 min compared to 43 min in their work as shown in Table 3. To demonstrate robustness and stability of our method, we added noise with different SNRs to the seismic data during testing to perform inversion. The inference time per seismic inversion on the Raspberry Pi was 22 s compared to 2 s on their Tesla GPU. Even though, our Raspberry Pi has very limited resources compared to their Tesla GPU, the inference times are very comparable and the difference is not much. Moreover, since we perform noise-aware training in our work, our models are able to generate good reconstructions even at low SNR noisy data compared to the ground truth, however we argue that their method will fail to generate good reconstructions if we feed our low SNR noisy data to their trained network. One of main contributions of our work is the execution of the inference on the resource-limited Raspberry Pi. Our proposed system is very portable and hence can provide real-time inversion results for data operators collecting data on the field in real world applications. Our designed user-friendly and interactive GUI also enables real time processing of the seismic data, fast model execution and generation of user-defined inversion results. Figure 13 shows the comparison of some velocity inversion results from our work and [25]. From the results, we can observe that our models (both no-noise and noise-aware) are able to generate better reconstructions compared to the results in [25].

## 8. Conclusions

In conclusion, we have proposed a novel edge-computing technique to perform seismic inversion on portable resource-constrained edge devices using the supervised data-driven based framework. Specifically, we implemented and executed DCN models to perform velocity inversion accurately, efficiently and practically on the edge device. Our DCN models consist of encoder and decoder structures built using layers of convolution and deconvolution. We used modified versions of the UNet and InversionNet models. We made great efforts to execute both UNet (shallow) and InversionNet (deep) models and performed inversion on the Raspberry Pi with very limited resources whilst achieving very feasible and acceptable inference time per seismic inversion. Even though, the training phase is computationally expensive, the inference time on Raspberry Pi for UNet and InversionNet models were 22 s and 4 s respectively which is very comparable to performing inversion on bulky GPU computers in [25]. We also demonstrated the robustness of our models through the addition of additive noise with different SNRs by performing no-noise training and noise-ware testing, and noise-aware training and testing in which our models were able to achieve superior performance. We also compared our results to the existing work [25] where our models were able to generate better inversion results compared to their work. We have also designed a user-friendly and interactive GUI application to completely automate and control the model execution and inversion process on the Raspberry Pi.

## Figures and Tables

**Table 1:** Test performance achieved on the raspberry pi by UNet model on Salt data.

	No-Noise UNet		Noise-Aware UNet	
**Sample**	**PSNR**	**SSIM**	**PSNR**	**SSIM**
1	16.3088	0.4873	16.0833	0.5169
2	17.9765	0.5340	17.8331	0.5338
3	12.9452	0.5110	14.4647	0.4907
4	13.4861	0.2539	16.4363	0.2779
5	26.2139	0.5325	24.2624	0.4877
6	22.5901	0.5032	16.1032	0.4251
7	22.7110	0.4629	19.2977	0.4159
8	15.8107	0.5168	14.9872	0.4984
9	12.4938	0.4285	19.2041	0.3643
10	13.9382	0.5091	18.0979	0.5965

**Table 2:** Test performance achieved on the raspberry pi by InversionNet model on Kimberlina data.

	No-Noise InversionNet	Noise-Aware InversionNet
**Sample**	**SSIM**	**SSIM**
1	0.984	0.991
2	0.999	0.999
3	0.978	0.981
4	0.983	0.983
5	0.999	0.999
6	0.993	0.992
7	0.995	0.999
8	0.942	0.961
9	0.999	0.969
10	0.997	0.998

**Table 3:** Comparison of training and inference times on Raspberry Pi, GPU PC and Yang and Ma [25].

Specifications	Hardware		
	Raspberry Pi 4 Model B	GPU NVIDIA GeForce GTX 1600 Super	Yang and Ma [25] HP Z840 workstation
Processor	Quad core—A72 1.5 GHz	Intel core i7 1.9 GHz	32 Core Xeon CPU
RAM	8 GB DDR4 SDRAM	16 GB DDR4	128 GB
Storage	Micro-SD (128 GB)	SSD (512 GB)	-
	Training time		
UNet	-	1 h 30 min (no-noise) 8 h (noise-aware)	43 min
	Inference time per prediction (s)		
UNet	22 s	2 s	2 s
InversionNet	4 s	18 s	-

## Data Availability

The two datasets used in the experiments were provided by the Earth and Environmental Sciences (EES) department of Los Alamos National Laboratory. The datasets can be released upon approval from Los Alamos National Laboratory and U.S. Department of Energy.
